# Intracoronary and tailored vasodilator therapy for refractory ventricular fibrillation storm triggered by multivessel coronary spasm: a case report

**DOI:** 10.1093/ehjcr/ytag506

**Published:** 2026-07-09

**Authors:** Aya Ishigaki, Masashi Koga, Yukio Sato, Yasuhiro Tanabe, Yoshihiro J Akashi

**Affiliations:** Department of Cardiology, St. Marianna University School of Medicine, 2-16-1 Sugao, Miyamae-ku, Kawasaki 216-8511, Japan; Department of Cardiology, St. Marianna University School of Medicine, 2-16-1 Sugao, Miyamae-ku, Kawasaki 216-8511, Japan; Department of Cardiology, Sagamihara Kyodo Hospital, 4-3-1 Hashimoto-dai, Midori-ku, Sagamihara 252-5188, Japan; Department of Cardiology, St. Marianna University School of Medicine, 2-16-1 Sugao, Miyamae-ku, Kawasaki 216-8511, Japan; Department of Cardiology, St. Marianna University School of Medicine, 2-16-1 Sugao, Miyamae-ku, Kawasaki 216-8511, Japan; Department of Cardiology, St. Marianna University School of Medicine, 2-16-1 Sugao, Miyamae-ku, Kawasaki 216-8511, Japan

**Keywords:** Vasospastic angina, Multivessel coronary spasm, Refractory ventricular fibrillation, Case report

## Abstract

**Background:**

Multivessel vasospastic angina (VSA) is a recognized cause of malignant ventricular arrhythmia. Although guideline-directed vasodilator therapy and implantable cardioverter-defibrillators (ICDs) are recommended, some patients remain at risk for refractory ventricular fibrillation (VF). To date, no strategy has been established for these cases.

**Case summary:**

A 67-year-old man experienced a VSA-related ST-elevation myocardial infarction complicated by VF, leading to subcutaneous ICD implantation. The patient remained stable for 1 year on medications without non-adherence or device therapies. One year later, the patient experienced out-of-hospital cardiac arrest due to a recurrent VF storm. Coronary angiography revealed intact arteries with diffuse spasms. Despite mechanical circulatory support, VF persisted and was unresponsive to repeated ICD shocks and external defibrillation. Repeat coronary angiography confirmed multivessel spasm. Intracoronary vasodilator administration, followed by defibrillation-terminated VF and subsequent tailored optimization of oral vasodilator therapy, prevented recurrence.

**Discussion:**

This case demonstrates that intracoronary vasodilator therapy combined with personalized drug adjustment may represent a valuable therapeutic option for refractory VF storms in multivessel VSA beyond conventional management.

Learning points

- Multivessel vasospastic angina (VSA) can trigger refractory ventricular fibrillation (VF) storms despite appropriate medical and device therapy.
- Intracoronary vasodilator administration can be life-saving in the acute phase by rapidly relieving diffuse coronary spasm and terminating defibrillation-resistant VF.
- Intensive and individualized long-term vasodilator therapy is essential to prevent recurrence in high-risk patients with VSA.

## Introduction

Vasospastic angina (VSA) is characterized by transient coronary artery spasm that may precipitate not only angina but also malignant ventricular arrhythmias (MVAs) and sudden cardiac death.^1,2^ Standard therapies for VSA include calcium-channel blockers (CCBs) and nitrates as guideline-directed vasodilator treatments, while implantable cardioverter-defibrillators (ICDs) are considered for secondary VSA prevention in patients with ventricular fibrillation (VF).^3^ However, recurrent VF can still occur, particularly in patients with severe or extensive coronary vasospasm, and optimal management strategies for such refractory cases remain poorly defined. Here, we report a case of refractory VF storm triggered by multivessel coronary spasm successfully stabilized using acute intracoronary vasodilator therapy followed by tailored long-term optimization of vasodilator treatment.

## Summary figure

**Figure:**
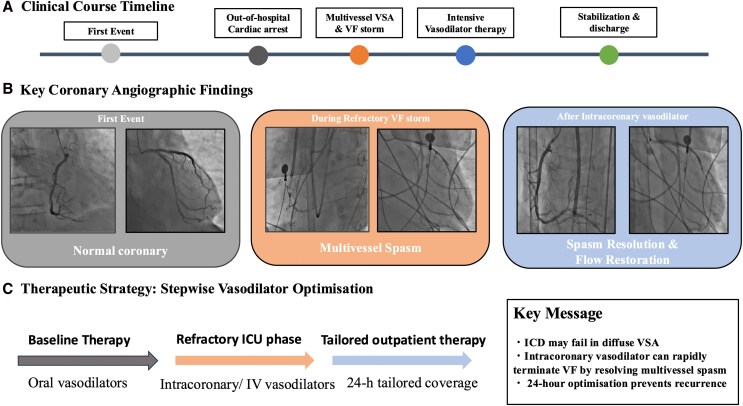


## Case presentation

A 67-year-old male with no significant medical history, family history, or cardiovascular risk factors developed an ST-elevation myocardial infarction during hospitalization for COVID-19 pneumonia. Electrocardiography revealed ST-segment elevation in Leads II, III, aVF, V5, and V6 (*Figure 1*). Shortly thereafter, the patient experienced VF, which was successfully terminated with a single defibrillation shock. The patient was transferred to our hospital, where emergency coronary angiography (CAG) was performed ∼12 h after symptom onset; CAG revealed no significant stenosis (*Figure 2A*, *E*). Provocative testing was not performed because of acute-phase haemodynamic instability and high risk of inducing recurrent MVAs. Intensive vasodilator therapy (dual CCBs and long-acting nitrate) was initiated as guideline-directed medical therapy to suppress recurrent coronary spasm. Diltiazem 100 mg/day and benidipine 4 mg/day were administered as a dual CCB therapy, along with isosorbide mononitrate 40 mg/day to achieve maximum pharmacological control.

**Figure 1:**
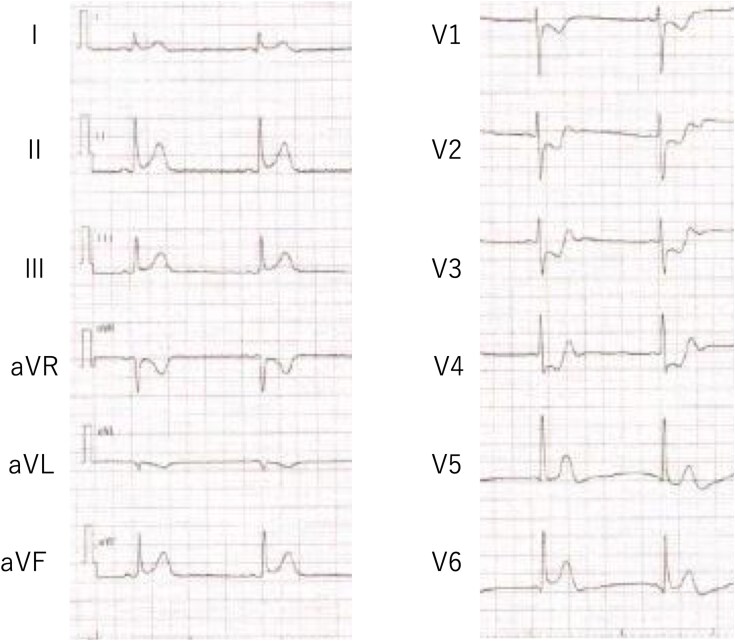
The 12-lead electrocardiogram at presentation. ST-segment elevation is observed in Leads II, III, and aVF; V5 and V6 demonstrate reciprocal ST depression in the anterior leads.

**Figure 2:**
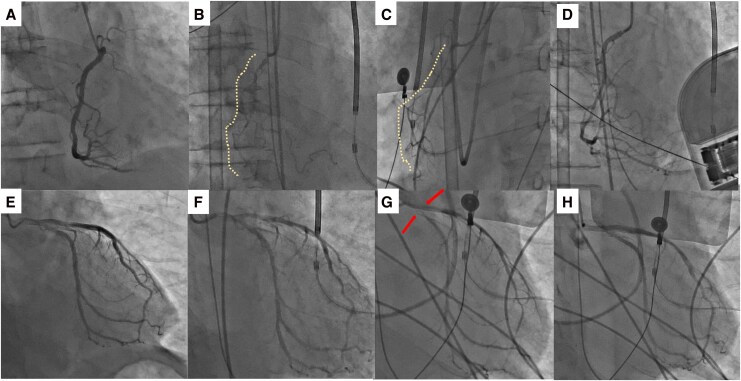
Coronary angiography. *(A)* Left anterior oblique (LAO) view of the right coronary artery at first admission, with no significant stenosis. *(B)* LAO view of the acute coronary spasm in the right coronary artery (dashed line). *(C)* Repeat angiography performed during refractory VF storm reveals a diffuse, severe spasm in the right coronary artery (dashed line, LAO view). *(D)* LAO view showing resolution of right coronary spasm after intracoronary vasodilator administration. *(E)* Right anterior oblique (RAO) caudal view of the left coronary artery at first admission, with no significant stenosis. *(F)* RAO caudal view with unobstructed left coronary arteries. *(G)* Repeat angiography performed during refractory VF storm reveals a diffuse, severe spasm involving the proximal left anterior descending and circumflex arteries (arrow, RAO caudal view). *(H)* Following intracoronary administration of a vasodilator, the left coronary spasm is promptly resolved, and normal coronary flow is restored.

Given the occurrence of VF associated with spontaneous ST-segment elevation in the absence of obstructive coronary disease, the patient was considered at high risk for recurrent MVAs. Therefore, a subcutaneous ICD (S-ICD) was implanted for secondary prevention in accordance with contemporary guideline recommendations.^3^

The patient remained clinically stable for 1 year, with no anginal symptoms, no evidence of non-adherence, and no ICD therapies. Approximately 1 year later, the patient complained of epigastric discomfort and visited a local clinic where he collapsed. At the local clinic, the initial documented rhythm was VF. Cardiopulmonary resuscitation and defibrillation achieved return of spontaneous circulation after one cycle of advanced cardiovascular life support. The patient was transferred to our hospital. On arrival, the patient was haemodynamically stable (blood pressure 120/74 mmHg, heart rate 84 b.p.m.) and neurologically intact. However, shortly thereafter, he developed recurrent VF with profound haemodynamic collapse, and his blood pressure became unobtainable despite advanced resuscitative measures. Bedside transthoracic echocardiography demonstrated minimal cardiac motion without pericardial effusion. Serum lactate increased from 2.2 mmol/L on arrival to 4.2 mmol/L immediately before mechanical circulatory support. Given the inability to maintain adequate circulation and end-organ perfusion, veno-arterial extracorporeal membrane oxygenation (VA-ECMO) and intra-aortic balloon pump (IABP) support were initiated immediately. Cardiac biomarkers were elevated, and a peak high-sensitivity troponin I level of 7.9 ng/L was observed. CAG performed immediately after resuscitation showed a severe diffuse spasm of the right coronary artery without fixed stenosis, while the left coronary system remained angiographically normal (*Figure 2B*, *F*).

These findings suggested recurrent coronary vasospasm similar to that observed during the initial event. Continuous intravenous administration of nicorandil and nitrate was initiated. Despite intravenous vasodilator therapy, recurrent diffuse ST-segment elevation was accompanied by progressive haemodynamic deterioration, with blood pressure declining to 68/34 mmHg. This was followed by recurrent VF that proved refractory to repeated ICD and external defibrillation shocks. Repeat CAG revealed severe multivessel coronary spasm involving all three major coronary arteries (*Figure 2C* and *G*). Given the patient's profound haemodynamic instability, intracoronary vasodilators were administered sequentially while carefully monitoring the haemodynamic response. Nitroprusside (50 μg), nicorandil (2 mg), and isosorbide dinitrate (1 mg) were administered in a stepwise manner, followed by successful defibrillation and restoration of sinus rhythm (*Figure 3*). The coronary spasm was relieved (*Figure 2D*, *H*), and the patient was admitted to the coronary care unit. During the acute phase, management included deep sedation, continuous infusion of diltiazem and nicorandil, and transdermal isosorbide therapy to prevent further spasm recurrence. He was successfully weaned off VA-ECMO on Day 5, and the IABP was removed on Day 6. The patient was successfully extubated on Day 12 without recurrent spasms. During step-down care, oral vasodilator therapy was carefully titrated to ensure continuous 24-h vasodilator coverage. The final regimen consisted of nicorandil 15 mg/day divided into three doses, isosorbide dinitrate 40 mg/day divided into two doses (morning and bedtime), diltiazem 60 mg/day divided into two doses, nifedipine controlled-release 20 mg at bedtime, nifedipine sustained-release 20 mg in the morning, and atorvastatin 10 mg daily. Device interrogation confirmed that S-ICD shocks were delivered during the VF storm but were ineffective. Considering this finding and the patient’s initial bradyarrhythmic presentation, the device was upgraded from an S-ICD to a transvenous ICD. Before discharge, transthoracic echocardiography demonstrated a preserved left ventricular ejection fraction of 55%.

**Figure 3:**
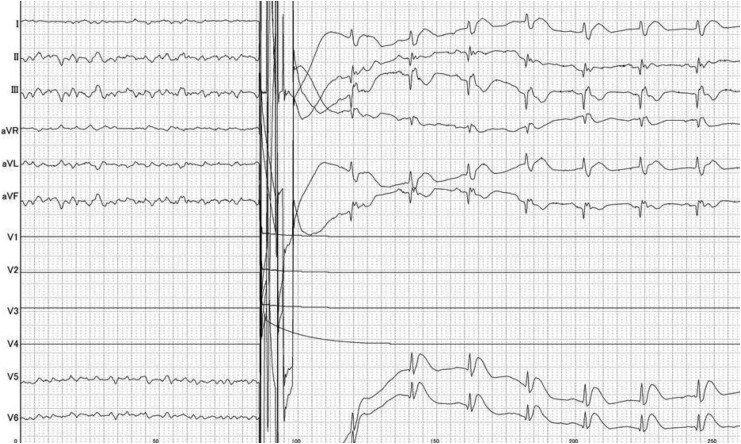
Electrocardiogram during CAG. VF is terminated by defibrillation after intracoronary vasodilator administration.

Despite a total post-cardiac arrest downtime of ∼39 min, no evidence of hypoxic brain injury was observed on brain computed tomography, and neurological recovery was favourable. The patient was ultimately discharged without neurological deficits. The patient recovered without further angina, arrhythmic events, or the need for ICD therapy. At the 12-month follow-up, the patient remained symptom-free with no recurrence of spasm or VF and regained an excellent quality of life.

## Discussion

This case highlights the challenges in managing MVAs associated with severe VSA, particularly when VF recurs despite guideline-directed vasodilator therapy and ICD protection. In this patient, diffuse multivessel coronary spasm resulted in extensive myocardial ischaemia, creating a highly unstable electrical substrate that predisposed to recurrent VF.

The initial event occurred during active COVID-19 infection, which has been associated with endothelial dysfunction and vascular hyperreactivity, possibly predisposing to coronary vasospasm; however, a causal relationship cannot be established in the present case.^4^ VSA is a recognized cause of MVAs, and recurrent VF may occur despite guideline-directed vasodilator therapy and ICD implantation.

Although ventricular arrhythmias associated with VSA may be considered potentially reversible with vasodilator therapy, contemporary guidelines state that ICD implantation may be considered in survivors of vasospasm-related cardiac arrest when the recurrence risk is considered high.^3^ In the present case, the initial presentation demonstrated several high-risk features, including VF causing out-of-hospital cardiac arrest and dynamic ST-segment elevation in the absence of obstructive coronary artery disease. These findings supported the decision to implant an ICD for secondary prevention. The subsequent documentation of diffuse multivessel coronary spasm during the recurrent event further supported the presence of a malignant vasospastic phenotype with a high risk of recurrent ventricular arrhythmias. Initially, an S-ICD was selected because the patient was young and had no bradycardia pacing indications. However, during the recurrent event, the patient presented with severe bradyarrhythmia and sinus arrest. Therefore, the device was upgraded to a transvenous ICD to provide not only defibrillation but also necessary bradycardia pacing and anti-tachycardia pacing support. The efficacy of the S-ICD for the prevention of sudden cardiac death has been shown to be non-inferior to that of transvenous ICD systems.^5^ Previous studies have shown that ICD shocks may fail to terminate VSA-related VF, leaving patients at persistent risk of recurrent arrhythmic events.^6,7^ In the present case, several pathophysiological mechanisms likely contributed to the refractory VF storm. First, diffuse multivessel coronary spasm caused extensive transmural ischaemia, creating a highly unstable electrical substrate. Global ischaemia promoted marked heterogeneity of myocardial repolarization, facilitating re-entrant electrical activity and perpetuation of VF. Second, the initial cardiac arrest and ongoing ischaemia likely triggered intense sympathetic activation, which may have further aggravated coronary vasoconstriction and lowered the threshold for ventricular arrhythmias. Third, prolonged ischaemia and circulatory collapse resulted in severe metabolic derangement, including intracellular acidosis, which likely increased the defibrillation threshold and reduced the effectiveness of repeated ICD and external shocks. In addition, underlying endothelial dysfunction and Rho-kinase hyperactivity may have predisposed the coronary arteries to diffuse and severe vasospastic responses. Successful termination of VF was achieved after intracoronary vasodilator administration and defibrillation, coinciding with angiographic resolution of severe coronary spasm and restoration of myocardial perfusion.

Within the spectrum of VSA, multivessel spasm represents a high-risk phenotype characterized by diffuse coronary involvement and worse outcomes than single-vessel disease.^2,8,9^ VF storms triggered by multivessel spasms are particularly difficult to manage, and outcomes in this context are generally poor.^9,10^ Several adjunctive strategies have been explored for refractory cases, including nicorandil,^11^ rho-kinase inhibition,^12^ and invasive procedures, such as cardiac sympathetic denervation.^13^ In selected patients with recurrent vasospastic events despite maximal medical therapy, combination vasodilator therapy, autonomic modulation, and individualized device-based strategies may all have complementary roles, although supporting evidence remains limited. In addition, acute-phase management often requires a multimodal approach to suppress the electrical storm. Deep sedation, employed in this case, is vital for reducing sympathetic drive, while intravenous magnesium may be considered for its electrophysiological stabilizing effects.^3^ Robust evidence supporting a standardized treatment strategy for acute VF storms in this population remains limited.

Intracoronary vasodilator administration for refractory coronary spasm has been described previously, particularly with nitroglycerine, demonstrating its potential to rapidly reverse severe vasoconstriction.^14^ Recently, intracoronary vasodilator therapy has been reported to successfully resolve diffuse multivessel coronary spasm complicated by cardiac arrest.^15^ However, the specific role of intracoronary vasodilator therapy in VF storm management remains poorly defined, and no standardized protocol has been established.

In addition to anti-spasm therapy, temporary mechanical circulatory support played an important role in stabilizing systemic perfusion during the period of profound haemodynamic collapse. In the present case, rapid progression from initial haemodynamic stability to refractory cardiogenic shock highlighted the fulminant nature of severe multivessel coronary spasm and provided a bridge to definitive treatment until coronary flow could be restored.

This case provided two important insights. First, VF storm can occur in multivessel VSA even under ICD protection and guideline-directed vasodilator therapy, underscoring current management limitations.^3,7^ Second, the acute intracoronary administration of vasodilators (nicorandil, isosorbide dinitrate, and nitroprusside) achieved rapid relief of coronary spasm, stabilized myocardial perfusion, and enabled successful VF termination by defibrillation. Subsequently, an intensive long-term vasodilator regimen, comprising dual CCB therapy, isosorbide dinitrate, and high-dose nicorandil, was optimized to provide continuous 24-h vasodilatory coverage and maximize the suppression of coronary hyperreactivity, which successfully prevented recurrence. A statin was included to improve endothelial function. Since oral rho-kinase inhibitors were not clinically available, we aimed to achieve a similar synergistic effect through this comprehensive pharmacological approach. Long-term management of malignant VSA remains challenging because recurrent vasospasm may occur despite apparently adequate medical therapy. In the present case, intensive combination vasodilator therapy was maintained to provide continuous anti-spastic coverage, and no recurrent ventricular arrhythmias were observed during follow-up. Careful longitudinal follow-up and ongoing ICD surveillance remain essential in these high-risk patients.

Study limitations include the absence of provocative coronary spasm testing, not performed because of the patient’s haemodynamic instability and the risk of inducing MVAs, thereby limiting formal diagnostic confirmation. Nevertheless, the diagnosis of VSA was supported by spontaneous angiographically documented multivessel coronary spasm with transient ST-segment elevation in the absence of fixed coronary obstruction. Notably, this electrocardiographic pattern was consistent with the patient’s initial presentation, where ST-segment elevation was observed in Leads II, III, aVF, V5, and V6 (*Figure 1*) despite no significant stenosis on the initial emergency CAG. These findings strongly support VSA as the underlying cause of the recurrent VF storms.

In conclusion, although this single-case report is hypothesis-generating and should be interpreted with caution, it highlights that severe multivessel coronary spasm may cause refractory VFdespite ICD protection and guideline-directed vasodilator therapy. In critically unstable patients with ongoing ischaemia and circulatory collapse, rapid recognition of refractory coronary spasm and aggressive multimodal treatment—including intracoronary vasodilator administration and mechanical circulatory support—may be life-saving. Further studies are needed to establish optimal management strategies for this high-risk population.

## Data Availability

The data underlying this article are available within the article. Additional data will be shared by the corresponding author on reasonable request.

## Lead author biography

Dr. Aya Ishigaki graduated from St. Marianna University School of Medicine in 2019 and completed her residency at the same institution. She subsequently undertook postgraduate training in cardiovascular medicine, with a clinical focus on coronary artery disease and vasomotor disorders.

## Notes

**Consent:** The authors confirm that written consent for the submission and publication of this case report, including images and associated text, was obtained from the patient in accordance with the COPE guidelines.
